# The potency of the butterfly: The reception of Richard B. Goldschmidt’s animal experiments in German sexology around 1920

**DOI:** 10.1177/0952695119890545

**Published:** 2020-06-04

**Authors:** Ina Linge

**Affiliations:** University of Exeter, UK

**Keywords:** animal studies, gender studies, German studies, history of sexuality, sexology

## Abstract

This article considers the sexual politics of animal evidence in the context of German sexology around 1920. In the 1910s, the German-Jewish geneticist Richard B. Goldschmidt conducted experiments on the moth *Lymantria dispar*, and discovered individuals that were no longer clearly identifiable as male or female. When he published an article tentatively arguing that his research on ‘intersex butterflies’ could be used to inform concurrent debates about human homosexuality, he triggered a flurry of responses from Berlin-based sexologists. In this article, I examine how a number of well-known sexologists affiliated with Magnus Hirschfeld, his Scientific-Humanitarian Committee, and later his Institute of Sexology attempted to incorporate Goldschmidt’s experiments into their sexological work between 1917 and 1923. Intersex butterflies were used to discuss issues at the heart of German sexology: the legal debate about the criminalisation of homosexuality under paragraph 175; the scientific methodology of sexology, caught between psychiatric, biological, and sociological approaches to the study of sexual and gender diversity; and the status of sexology as natural science, able to contribute knowledge about the sexual *Konstitution* of the organism. This article thus shows that butterfly experiments function as important and politically charged evidence for a discussion at the heart of the sexological project of those involved in the founding of the Institute of Sexology: the question of the nature and naturalness of homosexuality (and sexual intermediacy more broadly) and its political consequences. In doing so, this article makes a case for paying attention to non-human actors in the history of sexology.

In 1930, Magnus Hirschfeld published the fourth volume of his *Geschlechtskunde* series.^1^ Whereas volumes one to three, published between 1926 and 1930, focused on presenting a summary of Hirschfeld’s achievements in the area of sexology, the fourth volume is a *Bilderteil*, a volume focusing on sexology as represented through visual material. Amongst a wealth of images, it is the final one, printed on the very last page following the bibliography and a list of Hirschfeld’s publications, that holds a special place in this volume and, as I hope to show in this article, in the history of sexology more broadly. It is the image of the butterfly *Perrhybris lypera Magni Hirschfeldi* (Figure 1). The caption underneath the image notes that this species was named in honour of Magnus Hirschfeld, whose work was foundational to the scholarly discussion of transvestitism, a term he had coined in his publication *Die Transvestiten. Eine Untersuchung über den erotischen Verkleidungstrieb* (Transvestites: An Investigation Into the Erotic Drive to Cross-Dress) in 1910. As I want to argue in this article, debating sexual diversity with the help of non-human actors reveals a pressing sexological concern for the meaning of nature and naturalness for discussions of gender and sexual diversity.

**Figure 1.**
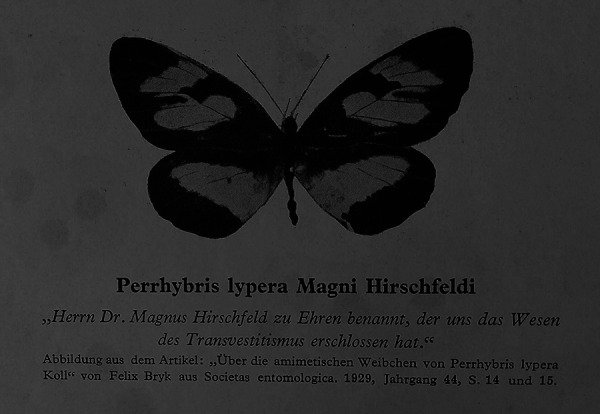
*Perrhybris lypera Magni Hirschfeldi* (Hirschfeld, 1930).

The butterfly in question was named after Hirschfeld by the Austrian-Swedish entomologist and anthropologist Felix Bryk in a 1929 issue of the German entomology journal *Societas entomologica*. Bryk worked predominantly on lepidoptera, but also published works in the area of ‘sexual ethnology’ with a focus on sub-Saharan Africa (see Schrader, 2019). The image of the butterfly in this special position, as bookend to the volume, underlines the purpose of the series to celebrate and immortalise Hirschfeld’s oeuvre and his fame in the area of sexual intermediacy, a term he coined in his *Zwischenstufenlehre* (study of sexual intermediacy), which claims that *Geschlecht* (sex/gender) can appear anywhere on a continuous scale between the opposing poles of male and female. This scale includes what we would today consider sexual as well as gender identities. However, the specific relationship implied by *Perrhybris lypera Magni Hirschfeldi* – between Hirschfeld’s work on sexual intermediacy, including transvestitism, and butterflies – is far from coincidental.

Over a decade before *Perrhybris lypera Magni Hirschfeldi* made an appearance in *Geschlechtskunde*, the German-Jewish geneticist Richard Benedict Goldschmidt (1878–1958) was conducting research on the gypsy moth *Lymantria dispar*. In his research of the early 1910s, Goldschmidt discovered that cross-breeding geographically distinct types of *Lymantria dispar* resulted in individual moths who were no longer clearly identifiable as male or female. He used the term ‘intersex’ to describe these individuals, and published an article tentatively arguing that this research could be used to inform concurrent debates about human homosexuality. His article triggered a flurry of responses in the *Jahrbuch für sexuelle Zwischenstufen* (Yearbook of Sexual Intermediacy), a journal edited by Magnus Hirschfeld that had become the mouthpiece of German sexology and was affiliated with the work done at the Institute of Sexology. In the following article, I want to discuss the responses of a series of intellectual intermediaries who discussed Goldschmidt’s intersex butterflies in the context of Hirschfeld’s *Yearbook*, including Max Hodann, Eugène Wilhelm, Arthur Kronfeld, Arthur Weil, and Hirschfeld himself. These men came from a variety of disciplines, from medicine to law and psychiatry, but shared a common interest in sexology. All of them referred to Goldschmidt’s butterfly experiments to discuss issues at the heart of German sexology: the legal debate about the criminalisation of homosexuality under paragraph 175; the scientific methodology of sexology, caught between psychiatric, biological, and sociological approaches to the study of sexual and gender diversity; and the status of sexology as natural science, able to contribute knowledge about the sexual *Konstitution* of the organism.^2^

In ‘“Unnatural Acts” in Nature: The Scientific Fascination With Queer Animals’, Jennifer Terry examines the ways in which geneticists, primatologists, biologists, and popular culture observe same-sex behaviour in animals. She asks, ‘What possibilities do animals, behaving in a “queer” manner, open up for humans interested in making sense of sexuality?’ (Terry, 2000: 152). This question is central to my work on the role of animals in the development of sexological debate, too, although the emphasis in sexological responses to Goldschmidt’s butterfly research is less on behaviour than on discussions of homosexual constitution, a sense of *being* homosexual, be it in a physical or a psychological sense. Terry’s article is important because it shows how animal evidence becomes an important vehicle for thinking about human queer desire and sexual behaviour that defies expectations of normative heterosexuality. Importantly, Terry’s article reveals the variety of ways in which, in scientific considerations of sexuality in nature, ‘the traffic between nature and culture’ (ibid.) is not universally regulated in its direction. This is important because, as Stacy Alaimo has argued, ‘much queer theory has bracketed, expelled, or distanced the volatile categories of nature and the natural, situating queer desire within an entirely social, and very *human*, habitat’ (Alaimo, 2010: 51). Paying attention to the ways in which non-human animals have historically been incorporated into models of queerness therefore makes an important contribution to understanding how the more-than-human world has been mobilised to shape concepts of sex, gender, and sexuality. My article further historicises what Jennifer Terry calls ‘the scientific fascination with queer animals’, and examines how sexological discussions about the nature and naturalness of homosexuality mobilised and politicised animal evidence, asking: under what conditions and for whom did the sexual indeterminacy of a species of moths come to matter for human sexual politics in interwar Central Europe?

To put sexologists’ interest in intersex butterflies in context, Goldschmidt’s butterflies/moths were not the only example of the political authority of animal nature in interwar sexology. The interconnected web binding together sex, development, and hormones featured prominently in the work of the Austrian endocrinologist Eugen Steinach. Steinach dedicated himself to the importance of the inner secretions (since 1905 also referred to as hormones) for the study of sex. He hypothesised that ‘the characteristics of sex could always be modified by modulating the functions of the sex glands’ (Sengoopta, 2006: 65). Steinach’s experimental work with guinea pigs and rats focused on same-sex and cross-sex transplants of gonads and concluded that the inner secretions produced masculine and feminine characteristics, arguing that sexuality was flexible and changeable rather than given in the gametes (Logan, 2013: 30–3). Hirschfeld took notice of Steinach’s work, and adopted his glandular theory of homosexuality to support his biological model of homosexuality as non-pathological and organic (Sengoopta, 1998). Hirschfeld showed a great interest in Steinach’s animal experiments, arguing that ‘nature had already produced human beings with the features of Steinach’s feminized and masculinized animals’ (ibid.: 464). As Sengoopta argues, Hirschfeld here used Steinach (and his experimental animals) as political allies for his sexual rights programme (ibid.). Steinach in turn drew on Hirschfeld’s clinical work to argue that lacking gonadal differentiation caused homosexuality. Convinced by Hirschfeld that his experimental work could be applied to humans, Steinach ‘turned enthusiastically from guinea pigs to men’ (ibid.: 465), and collaborated with the urologist Robert Lichtenstern on gonadal grafting in humans (Oudshoorn, 1994: 57). Hirschfeld and his colleagues adopted this practice. For Hirschfeld, Steinach’s animal experiments confirmed that homosexuality was indeed of a biological nature, with direct political implications: homosexuality was part of a natural spectrum of sexuality and could not be criminalised.

Sexologists evidently had an interest in animal evidence more broadly, but it is important to keep in mind the specificity of the particular animal used as a form of evidence for human sexuality. Whereas some geneticists, such as Thomas Hunt Morgan, built their experimental work on *Drosophila* (the fruit fly) as a model organism, Goldschmidt built his experimental work on the gypsy moth *Lymantria dispar*. As Michael Dietrich has shown, the choice of model organism used by scientists studying sex determination had a profound impact on the interpretation of results. Dietrich examines the experimental systems of four scientists in relation to their model organisms – Tatsuo Aida working with the small fish medaka, Øjvind Winge with guppies, Calvin Bridges with the fruit fly, and Goldschmidt with the gypsy moth – arguing that ‘the interpretive differences between Aida, Bridges, Goldschmidt and Winge were rooted in differences within their experimental systems that each constituted sex and sex determination in substantially different ways’ (Dietrich, 2016: 38). As a result, this article traces the use of butterflies specifically, not simply as a model organism modelling generalisable biological processes, but importantly also as one offering specific possibilities and parameters to Goldschmidt’s experimental system and to the meaning assigned to butterfly evidence by sexologists around 1920.

In a post-Darwinian context, the marshalling of animal evidence by sexologists to explain human conditions is not surprising, but what kind of evidence is used, the framework of thought implied, and to what ends it is used can have political implications. In *Looking for a Few Good Males*, Erika L. Milam shows how biologists in the early 20th century, for example, used Darwin’s theory of sexual selection, which Darwin had conceived of primarily to explain the concept of beauty in the animal kingdom, to frame female choice in the language of mechanistic stimulation (Milam, 2010: 2). Using animal evidence to make sense of human relations in this way has different consequences for sexual politics than, for example, using sexual selection with a focus on sexual agency (ibid.). The particular ‘moral authority of nature’, as discussed by Lorraine Daston and Fernando Vidal (2004), is revealed here as influencing political views on gender and sexuality via non-human animals. If nature has a moral authority, then seeing sexual diversity and equality of sexes in the animal kingdom becomes important. As Kirsten Leng argues, early 20th-century female and feminist sexologists in Germany and Austria utilised the authority of nature through references to the animal kingdom to argue that their feminist demands were in line with nature’s intent (Leng, 2018). Nonetheless, as Milam argues, ‘despite the long history of using animals as models of human behaviour, the idea that we could learn about ourselves from studying fruit flies or guppies was far from intuitively obvious and required intellectual work’ (Milam, 2010: 7).

It is this intellectual work that I want to examine in this article: How was knowledge about butterflies transferred to humans and to what ends? What scientific and methodological frameworks were used to facilitate this transmission? One aspect of this intellectual work was the transmission of evidence across disciplinary lines: Using the experimental work on intersex butterflies in the context of sexological work required a translation from one disciplinary context to another, from zoology and animal genetics to sexology. By paying attention to this interdisciplinary exchange between animal genetics and sexology, this article also builds on recent work on the interdisciplinary nature of sexology (Fisher and Funke, 2015, 2018). Tracing this cross-disciplinary and often interdisciplinary exchange of evidence shows that the meaning and significance of butterfly evidence was not fixed, as evidence had to be fitted into an already existing but continually developing set of sexological hypotheses and methodologies. The circulation of these claims amongst sexologists put them into a different sociopolitical context, with ramifications for how they could be understood both scientifically and politically. The necessity of marshalling butterfly evidence lay in the fact that particular authority was accorded to natural and biological science. By relying on evidence from animal genetics, sexologists thereby also used this cross-disciplinary knowledge transfer to support sexological theories and widen their field of influence. In the following article, then, I explore the complex politics of evidence that took place when intersex butterflies were used to inform central sexological questions, most importantly the debate around the nature and naturalness of sexual intermediacy.^3^

## Sex and race in intersex butterflies: Richard B. Goldschmidt

German-Jewish geneticist Richard Benedict Goldschmidt pioneered research on intersex butterflies. Goldschmidt studied medicine and zoology, and in 1914 was appointed as the head of his own department, focusing on animal genetics and biology, at the new Kaiser Wilhelm Institute (KWI) for Biology in Dahlem, Berlin (Goldschmidt, 1960: 79–81). Goldschmidt’s research depended on access to geographically diverse specimens of his experimental organism, the gypsy moth *Lymantria dispar*, and therefore took him to Japan several times: in 1914, for two years from 1924 to 1926, and a final time in 1929 (Goldschmidt, 1960). Intending to return to Germany via a research stay in the United States after his first Japan trip, he was caught up in the outbreak of World War I. Unable to return to Germany, he secured a position as visiting scholar at the Osborn Zoological Laboratory at Yale University (Richmond, 2015: 53). Increasing war hysteria in the USA led to accusations that Goldschmidt was a pro-German spy, and in May 1918 he was interned at Fort Oglethorpe, Georgia, a detention facility for ‘dangerous enemy aliens’ (ibid.: 59). Goldschmidt finally took up his post at the KWI in 1919 and stayed there until 1935, when he was dismissed from his position by Nazi decree. He returned to the USA and settled in California, where he became professor of zoology at the University of California, Berkeley, retiring in 1948 (ibid.: 62).

Goldschmidt’s work was part of a larger scientific effort of biologists to understand the nature of sex. In *Sex Itself*, Sarah Richardson (2013) gives a comprehensive account of the history of scientific discoveries and theories of sex that I will outline here briefly. In the 1850s, Rudolf Virchow’s discovery that all tissues arise from a single cell cast the cell as the basis of life. Complemented by Charles Darwin’s theory of evolution, biological research on heredity – ‘how traits are transmitted and recombined through sexual reproduction’ (ibid.: 27) – were imbued with new life. In the second half of the 19th century, cytologists tried to locate the hereditary units in the cell, and reproductive biology became the main site of investigation. From the 1890s onwards, chromosomes became candidates for the physical basis of heredity; recently discovered X-shaped elements were hypothetically linked to sex. American cytologists Nettie M. Stevens and Edmund B. Wilson insisted on the relevance of the X-shaped (and after Stevens’ discovery, the Y-shaped) chromosomes for a theory of heredity, but disagreed on how these ‘odd chromosomes’ related to sex determination. The question remained: by what mechanism could these chromosomes determine sex?

In the midst of these debates, the significance of Mendel’s laws of heredity, first developed in the 1860s, was rediscovered by biologists. Mendel’s experiments on pea plants showed that individual traits are determined by pairs of factors (alleles) that separate during gamete formation and randomly recombine, producing genetic diversity. In the early 1900s, chromosomes were suspected of being these carriers of genetic material. The ‘odd chromosomes’ discovered by Stevens and Wilson were increasingly called ‘sex chromosomes’. In the early decades of the 20th century, geneticists like Goldschmidt now set out to develop theories of the gene to investigate what aspect of the sex chromosomes determined sex from one generation to another, and whether this took place according to Mendelian laws of heredity. In the late 19th century, Richardson asserts, ‘researchers…believed that sex was a continuous (spectrum) rather than a discontinuous (binary) trait’ (Richardson, 2013: 24). By the 1930s, sex chromosomes had been drawn into the centre of molecular biology, and ‘today’s notion of the X and Y as the molecular agents of sex and as the genetic homunculi underlying sexual dimorphism—as “sex itself”—[was] first recognizable’ (ibid.: 71). But during Goldschmidt’s early years and the early years of genetics leading up to this moment, hormonal and genetic sex determination was considered complex and fluid.

Goldschmidt was an influential, prominent, but also controversial figure in the nascent field of genetics arising in this context of sex research. Michael Dietrich has written extensively about Goldschmidt’s ‘heresies’ in interwar genetics, which questioned the orthodoxy of the American evolutionary biologist Thomas Hunt Morgan (1866–1945). Whereas Morgan focused on gene transmission alone, Goldschmidt’s theory focused on quantitative and physiological aspects, and on gene action and function (Dietrich, 1995: 435). In the 1930s, his work underwent a major change as he began to argue that the chromosome, rather than the gene, was the unit of heredity (ibid.: 436). This dissenting view led to serious criticism of Goldschmidt’s work and his standing as an influential figure in genetics. Dietrich argues that Goldschmidt’s reputation as iconoclast was cemented by the response to his 1940 book *The Material Basis of Evolution*, in which Goldschmidt criticised a widely accepted theory of neo-Darwinian evolution based on gradual change, arguing instead that evolutionary change required the existence of ‘hopeful monsters’, complete changes caused by macroevolution. Dietrich shows that although Goldschmidt’s idea is mostly rejected today, it engendered significant interest immediately after publication (ibid.: 432–3).

**Figure 2.**
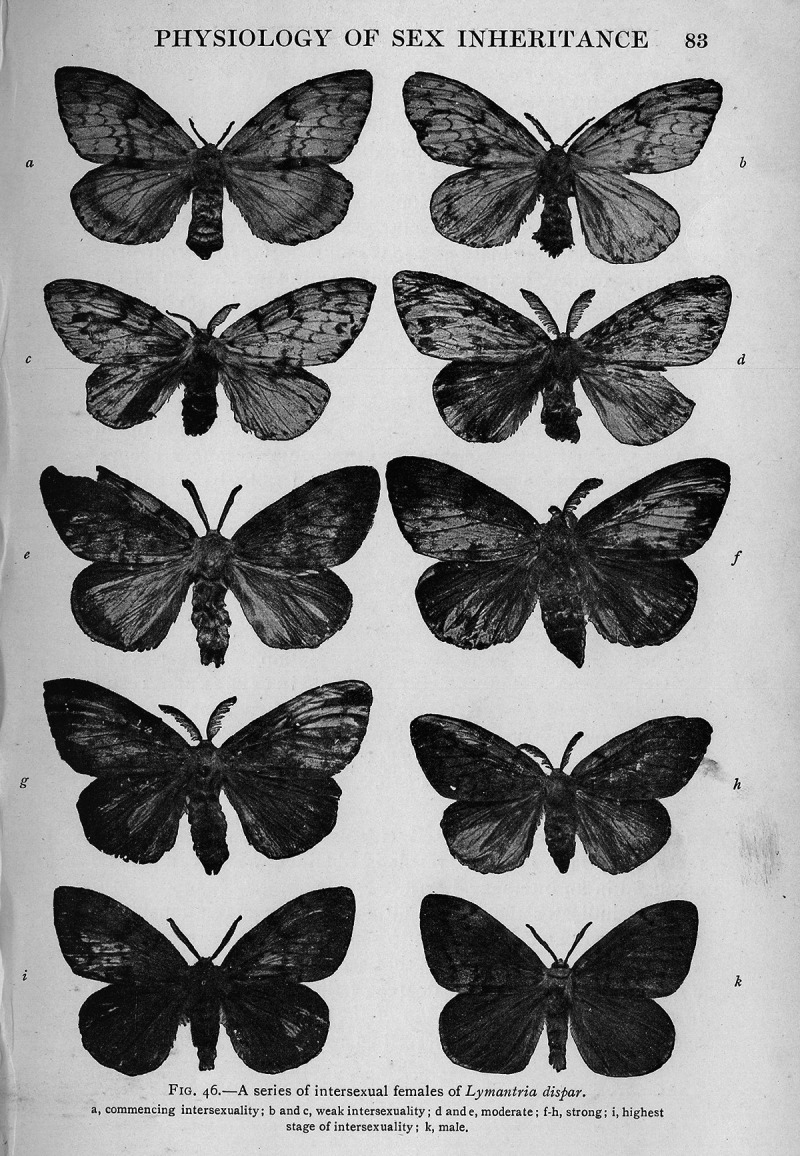
A series of intersexual females of *Lymantria dispar* (Goldschmidt, 1923: 83).^11^

Goldschmidt’s experimental work of the early 1910s investigated the heredity of sex difference using different ‘races’ of the gypsy moth from Japan and Europe.^4^ ‘Race’ here refers to geographically distinct populations of an animal species (Goldschmidt, 1915: 565). Certain combinations of different populations resulted in offspring that were no longer sexually dimorphic and displayed intermediate sexual characteristics (Dietrich, 2016: 26). Rather than combining male and female characteristics, however, these individuals were shown to have developed initially as female and then developed as male later on, and vice versa (Richmond, 2007: 177). As a result, Goldschmidt called these individuals ‘intersex’. Goldschmidt hypothesised that intersexuality was caused by a difference in ‘potency’ or ‘valence’. According to Goldschmidt, each organism contained the predisposition for either sex. Within each organism, a quantitative value of the sex factor –– its ‘valence’ or ‘potency’ –– and its relation to the potency of the predisposition of the other sex, decided which one finally appeared (Goldschmidt, 1916: 709). Goldschmidt argued that the quantitative values of the sex factor varied across geographical regions. He therefore used various natural populations of the gypsy moth from different geographic regions in Europe and Japan in order to study sex expressions in genitalia, antennae, colouration of the wings, and so on, all of which were particularly distinct in female and male individuals of *Lymantria dispar* (Satzinger, 2009a: 252–4). This made representations of intersex individuals of the gypsy moth particularly striking (see Figure 2). It was this experimental research based on the cross-breeding of different populations of the gypsy moth that was reviewed extensively by sexologists around 1920.

Scientific discussions of the sex problem were closely embedded in the political, social, and cultural circumstances of the early 20th century. They were informed by and informing broad debates about eugenics, feminism, and theories of race and ‘miscegenation’.^5^ Koehler and Satzinger show that Goldschmidt’s scientific discussion of sex determination, and the wider context of Goldschmidt’s colleagues and rivals, was profoundly tied up with the larger social concern of modernity: a changing context of gender, gender relations, and sexuality, in particular homosexuality (Koehler, 1998: ii; Satzinger, 2009b: 159). Goldschmidt’s work, which, he argued, showed that there was no clear-cut two-sex system, but that sexual difference existed on a sliding scale, was particularly pertinent in the Weimar Republic, where the social order of gender was rapidly evolving (Satzinger, 2009b). In this context, Goldschmidt’s butterfly experiments were co-opted for various political uses. Whilst this article focuses on the ways in which Goldschmidt’s butterflies were made to serve sexological arguments about human sexual diversity, his work was also incorporated into specifically right-wing and *völkisch* political agendas. Satzinger has shown that geneticist and eugenicist Fritz Lenz used Goldschmidt’s work to argue that interracial sexual contact was the cause of degeneration, because it supposedly led to the erosion of a clear sex binary (ibid.). Dietrich shows that the physician Theo Lang, a supporter of National Socialism, used Goldschmidt’s early comments on the genetic foundation of homosexuality to suggest sterilisation as a ‘solution’ to the ‘problem’ of homosexuality (Dietrich, 2000).^6^

As a consequence, sexologists’ interest in intersex butterflies has to be considered as part of the broader attempts to accommodate Goldschmidt’s work in very different models of race, gender, and sexuality. In 1916, Goldschmidt catapulted himself directly into these political discussions by publishing an article in which he argued that his butterfly experiments were of relevance to the study of human intersex, suggesting that homosexuality was one such form of intersex in humans (Goldschmidt, 1916/18).^7^ Although Goldschmidt’s work hardly ever commented on issues as politically charged as the topic of homosexuality, there are several reasons why he felt it appropriate to comment on this occasion. As Koehler argues, ‘Goldschmidt…sought to produce a single universal theory of sex.…Goldschmidt used his work on intersexuality to explain human homosexuality, and argued that the phenomena of sex were fundamentally the same’ (Koehler, 1998: 57–8). Goldschmidt chose to focus on (male) homosexuality as a form of intersex as the simplest choice from a selection of more difficult options (ibid.: 221). Beyond this simple suitability of the topic of male homosexuality, Koehler argues that Goldschmidt was frustrated with the likes of Hirschfeld, who lacked specialist knowledge in the biological sciences and genetics, weighing in on the sex question (ibid.: 198). Goldschmidt wrote, ‘there is hardly another problem which has been such a playground of dilettantism, and if we look through the older literature on the sex problem, we find almost as many philosophers and economists inventing sex-theories as there are biologists’ (Goldschmidt, 1916: 705). Dietrich understands Goldschmidt’s comment here as a reference to the ‘growing number of sex researchers and the new field of sexual science’, arguing that ‘Goldschmidt used the experimental nature of his research as a means both of claiming greater authority to speak on the science of intersexuality and of differentiating himself from Hirschfeld and others’ (Dietrich, 2000: 225).

In the process of writing his article about human homosexuality from the standpoint of animal genetics, Goldschmidt familiarised himself with sexological literature and contributed to an interdisciplinary debate about the nature of homosexuality. Researching for his article on human hermaphroditism whilst in the USA, he asked the director of the Eugenics Record Office, Charles Davenport, for assistance in finding copies of the *Yearbook of Sexual Intermediacy* (Dietrich, 2000: 224). The *Yearbook* was edited by Hirschfeld and published by the Wissenschaftlich-humanitäres Komitee (the Scientific-Humanitarian Committee, SHC), an organisation that campaigned to end legal prosecution of sexual and gender minorities. Originally, Goldschmidt understood human hermaphroditism as continuous with homosexuality, and presented the argument that homosexuality was not pathological and as irrelevant to health as colour-blindness (Satzinger, 2009b: 156). This argument was taken directly from the sexological context, as several sexologists, including Hirschfeld, the British sexologist Henry Havelock Ellis, and his co-author, the British literary critic John Addington Symonds, had previously argued that homosexuality could be compared to colour-blindness (Ellis and Addington Symonds, 2008[1896]: 204; Hirschfeld, 2001[1914]: 372–3).^8^ Goldschmidt also cited at length from Moll’s 1912 *Handbuch der Sexualwissenschaften* (Handbook of Sexology).^9^ He called Moll’s theory that sex was always a mix of male and female components ‘more or less vague’, and promised that his own research could provide experimental proof for Moll’s theoretical approach. Whilst this may have been a way of differentiating himself from Hirschfeld and Moll as sexologists, it also speaks to a certain level of interest in their findings and a concern for the same issues, which Goldschmidt merely approached from a different disciplinary standpoint, that of animal genetics. Goldschmidt became so familiar with sexological scholarship as to forward reprints of articles about homosexuality to the British embryologist Julian Huxley (Koehler, 1998: 226).

Despite his apparent conviction that the sex question was best left to biologists and geneticists, Goldschmidt, too, tentatively contributed to debates outside his immediate field of expertise, and spoke in favour of such interdisciplinary work. He opened his article on human hermaphroditism with the words:

The infinite specialization of science [*Wissenschaft*] naturally means that results of one branch of research often remain hidden from experts in a neighbouring field, even if they in fact concern identical problems, which are merely approached from different disciplinary angles. And that probability is often magnified by the fact that no conscientious scientist loves to inexpertly dabble in a field that he [*sic*] does not fully master. But numerous cases can be devised in which such encroachments are not only justified but indeed useful. (Goldschmidt, 1916/18: 1)^10^

In the following sections, then, it should be noted that sexological debates and Goldschmidt’s zoological work influenced one another. In what follows, I want to trace how Goldschmidt’s contribution to the study of human intersex via butterfly experiments was received by sexologists in the *Yearbook of Sexual Intermediacy*, in order to show how butterfly evidence was co-opted to make political arguments about the nature and naturalness of homosexuality. In doing so, this article will outline the sexual politics of butterfly evidence.

## The sexual politics of scientific practice: Max Hodann

In 1917, a review article by Max Hodann (1894–1946), entitled ‘Neue Forschungen zur Kenntnis der hereditär-physiologischen Grundlagen sexueller Zwischenstufen’ (New Research on the Hereditary-Physiological Foundations of Sexual Intermediacy), was published in the *Yearbook of Sexual Intermediacy*. In his article, Hodann took particular note of Goldschmidt’s 1916/18 article, which outlined the biological basis of contrary sexuality and hermaphroditism in humans on the basis of his experimental work on intersex butterflies. Hodann’s article was largely a very faithful summary of Goldschmidt’s argument. He cited at length from Goldschmidt’s article and paraphrased Goldschmidt’s main points, adding only his own brief introduction and concluding remarks. In this way, he lifted Goldschmidt’s work out of its context and placed it directly into a sexological journal, introducing these intersex moths to a sexological readership for the first time. As I want to argue in this section, Hodann’s article shows how scientific research on butterflies was marshalled to support a particular political vision and sexological practice, and was also used to achieve a certain level of professional prestige.

Hodann’s interest in sexology was sparked by an encounter with Hirschfeld at a talk he gave in 1915 (Herzer, 1985). The article about Goldschmidt’s intersex butterflies in the *Yearbook* was his first sexological publication, published after his first encounter with Hirschfeld two years previously. The article reflected Hodann’s enthusiasm for Hirschfeld’s work and its application in a clinical context. The complementary nature of Goldschmidt’s and Hirschfeld’s evidence was one of the main points Hodann tried to put across in his article. What Hodann found important about Goldschmidt’s article was that ‘it approaches the often painfully absent connection between natural-scientific and zoological laboratory work, and clinical observation’ (Hodann, 1917: 60). What he meant by this was that Goldschmidt’s work on butterfly intersexuality produced comparative results to the clinical model of sexual intermediacy. Butterfly experiments and clinical observations of human patients showed the same results.

If we accepted this, Goldschmidt argued (and Hodann agreed), several conclusions presented themselves. First, intersexuality arose from the crossing of geographically diverse individuals. Whereas Goldschmidt’s article went on to explain the complexity of this issue, arguing that human populations were always already ‘racially’ mixed – that is, geographically distinct (Goldschmidt, 1916/18: 8) – Hodann reduced this conclusion to the simple statement that ethnographic data might help the sexologist understand the phenomenon of human intersexuality (Hodann, 1917: 65). He reduced Goldschmidt’s complex argument and focused on the importance of ethnographic data for the study of sex in order to show the compatibility with Hirschfeld’s work. Hirschfeld showed a keen interest in ethnographic material (see Bauer, 2011; Fisher and Funke, 2018). Another conclusion that Hodann drew alongside Goldschmidt was that in humans, unlike insects, the inner secretions played an important role in sexual development (Goldschmidt, 1916/18: 12; Hodann, 1917: 67). Goldschmidt tentatively argued that ‘intersexuality could be “cured” through treatment with gonadal extract or transplantation of normal gonads’ (Goldschmidt, 1916/18: 14). Framing a cure in quotation marks suggests that Goldschmidt did not consider intersexuality/homosexuality a disease, and indeed the following conclusion confirmed this: An intersexual individual could not be assigned a sex, because they were not fully male or female (ibid.). Although he did not follow this with a statement about the decriminalisation of homosexuality, it is clear that Goldschmidt understood intersexuality as part of natural sexual variation. Hodann’s article also pointed out this ‘healing effect’ (‘Heilwirkung’; Hodann, 1917: 67) of extract and transplant therapy. Whereas Goldschmidt was less interested in the clinical application of his work, Hodann, whose work focused on the clinical application of sex research, took the potential for hormone and gonad treatment into serious consideration. Again, this was in line with Hirschfeld’s interest in these procedures in his search for a possible proof for the biological foundation of (homo)sexuality (Beachy, 2014: 175).

Hodann would have relied on Hirschfeld’s clinical material to inform his thinking, because, at the time of writing, he had not finished his medical degree and would not have had extensive clinical experience himself. In this way, Hodann attempted to establish both himself and sexology. In 1917, sexology was still in the process of establishing itself as a scientifically recognised field. Hirschfeld was less than two years away from founding his Institute of Sexology. Any cross-disciplinary link with the well-established and well-funded biological sciences could only help the sexological cause. In demonstrating his allegiance to Hirschfeld’s sexological theories by contributing a link with biological sciences, Hodann made a space for himself in sexological circles, both figuratively and literally speaking: In the mid-1920s, he joined the staff of the Institute of Sexology. Using Goldschmidt’s butterfly experiments from the field of animal genetics to support a hypothesis from the emerging field of sexology was therefore also a political move to support sexology’s credibility.

Hodann then asked what *legal* conclusions sexologists could draw from this, and directly cited Goldschmidt to answer this question for the sexologist:

Physical and psychological intersexuality of all stages exists. An individual that is genetically supposed to belong to one sex is, in fact, intersex. From this it follows that –– because laws cannot be based on chromosomes, but on the actual situation of an individual –– no court of law is able to assign sex/gender…because this individual belongs, in actual fact, to neither of the two sexes. (Goldschmidt, 1916/18: 14, cited in Hodann, 1917: 68)

Here, Goldschmidt’s remarks anticipated the work of much more recent feminist scholars working with biological arguments, for example Anne Fausto-Sterling. In her seminal article, ‘The Five Sexes: Why Male and Female Are Not Enough’, Fausto-Sterling argues that ‘if the state and the legal system have an interest in maintaining a two-party sexual system, they are in defiance of nature. For biologically speaking, there are many gradations running from female to male’ (Fausto-Sterling, 1993: 21).

To this, Hodann added his own conclusion: ‘Those who have had the opportunity to observe the sheer diversity of sexual intermediate types in life, as well as in physical and psychological manifestations, can only agree with Goldschmidt’s…conclusions’ (Hodann, 1917: 68). Here, he returned to the idea that experimental work on butterflies conducted in a controlled laboratory environment and clinical observation stood in dialogue, and showed comparable results. For Hodann, the natural diversity of sexual life – in both butterflies and humans – had political consequences. Whilst Hodann was establishing himself as an ally of sexological causes, he was also particularly interested in social hygiene, public medicine, and sex education, spurred on by his political affiliations with the German socialist movement. When he joined the staff at the Institute of Sexology in the mid-1920s, he did so in the capacity of sex educator/adviser (Sigusch and Grau, 2009: 296–302). He lectured on topics including abortion, contraception, masturbation, and sexuality. He also wrote sex education material from a socialist perspective specifically for working-class children (Sauerteig, 2009: 133–5; 2012). As Atina Grossmann argues, ‘Wherever the ubiquitous Hodann published, he always used his dual authority as physician and working-class activist to plead for tolerance’ (Grossman, 1995: 29). In his use of Goldschmidt’s experimental work on butterflies, Hodann referred to the natural diversity and variation of sex, which depended on ‘potency’, rather than a clear and definite appearance of sex, to argue that the law should recede, because it was not fit to categorise people into male and female. The argument was not that we should do what nature said (he explicitly said he did not want to assign sex according to chromosomes), but that the different layers of sex added up to a complex system that could not be adequately represented. Intersex butterflies were used to make the argument that natural variation exceeded its legal comprehension. This line of argument was in line with Hodann’s general approach to the scientific study of sex, which considered medicine and science as fundamentally political.

In Hodann’s review of Goldschmidt’s work, intersex butterflies were mirror images of sexual intermediates in the human realm. This was reflected by the larger disciplinary twinning Hodann conducted of Goldschmidt’s experimental work and Hirschfeld’s clinical observations. Both experimental and clinical evidence, Hodann argued, provided a natural basis for tolerance. In this way, the intersexuality of butterflies was marshalled to provide a basis for tolerance of sexual diversity in the human realm, and thereby also clarified Hodann’s position as advocator for Hirschfeld’s work, which would later secure him a position at the Institute. In Hodann’s case, butterfly evidence was political in a double sense: It was marshalled to support a political aim, namely the recognition of sexual diversity beyond a limiting two-sex model, and it was a political move to establish the emerging field of sexology as equal to the more established biological sciences.

## The science behind gay rights activism: Eugène Wilhelm

Three years after Hodann, Eugène Wilhelm (1866–1951) published another review of Goldschmidt’s article (Praetorius, 1920). Wilhelm was a lawyer and sexologist originally from France. He was not medically trained, but he had a significant influence on sexology because from 1900 to 1922 he wrote the ‘Bibliography of Homosexuality’, the review section of the *Yearbook*, under the pseudonym Numa Praetorius (Dubout, 2016: 57). The Bibliography reviewed several hundred publications, at times took up a third of the *Yearbook*, and covered a total of 2300 pages (ibid.: 58). Wilhelm published three reviews in the *Yearbook* in which he drew on Goldschmidt’s zoological research. As I will argue in this section, Wilhelm used Goldschmidt’s butterfly experiments to depoliticise nature, arguing instead that nature’s interpreters, especially those who opposed gay rights, were driven by retrograde and flawed political aims, and were motivated more by politics than by scientific knowledge.

In his review of Goldschmidt’s work from 1920, Wilhelm, like Hodann, faithfully summarised Goldschmidt’s argument, but where Hodann focused on the comparability of experimental and clinical work to show the natural variation of sexual intermediacy, Wilhelm was interested exclusively in the meaning of Goldschmidt’s work for the argument that homosexuality was inborn. Wilhelm argued that homosexuality was not acquired through external factors, but was deeply linked to the body’s earliest developmental stages. Goldschmidt’s research, he argued, proved this: Homosexuality was one form of intersex, and Goldschmidt’s butterfly experiments had shown that these were linked to an individual’s genetic predisposition. Whereas Hodann saw hereditary experiments on butterflies and clinical observation of human patients as producing comparable outcomes, when Wilhelm introduced Goldschmidt’s work, he did not mention that his model organism was the gypsy moth, but simply talked about intersex in general terms: ‘As such, when two races (*e.g. butterflies*) are bastardised, it should be possible to achieve…’ (Praetorius, 1920: 81; emphasis added). Butterflies here became an exemplary species, implying that these rules applied to all species, including humans.

For Wilhelm, Goldschmidt’s butterflies were a piece of evidence that fitted into an already existing hypothesis of the naturalness of homosexuality. Indeed, articles arguing for the naturalness and innateness of homosexuality took up the largest number of pages in Wilhelm’s review section, whereas the review section discussing homosexuality as acquired received far fewer contributions (Dubout, 2016: 60). The argument that homosexuality was inborn had long and deep roots: Karl Heinrich Ulrichs had already made the same claim several decades earlier (Ulrichs, 1870). Magnus Hirschfeld, too, was committed to finding scientific proof that homosexuality was inborn, and used this as a foundational argument for his gay rights activism. Hirschfeld’s double effort as initiator of sexological theories and methodologies, as well as gay rights activist, caused clashes with sexologists and scientists who proposed a different model of homosexuality, for example one based on sexual pathology.

What was particularly important about the argument that homosexuality was natural and inborn was that Wilhelm used this as proof that those who attacked Hirschfeld’s scientific endeavours as politically driven were in fact politically driven themselves. In another article, published in the *Yearbook* in 1922, in which he used Goldschmidt’s work as evidence, Wilhelm reviewed the work of the German psychiatrist Emil Kraepelin. Kraepelin was an influential psychiatrist, who argued that homosexuality was a form of psychopathology that could be avoided through education and by prohibiting the ‘promotion of homosexuality’ (‘homosexuelle Werbearbeit’; Kraepelin, 1918: 119, cited in Weber and Burgmair, 1997: 5). Wilhelm countered Kraepelin’s argument by saying that Kraepelin’s ‘theoretical and speculative arguments, which are influenced by moral and socio-political viewpoints and which are used here, naturally lead to skewed judgment’ (Praetorius, 1922: 42). This is particularly interesting as sexologists like Wilhelm and Hirschfeld were often criticised for being politically driven, because their scientific work was so closely linked to their activism to decriminalise homosexuality. Kraepelin had made this argument against Hirschfeld several times before, for example in a censorship report against *Anders als die Andern* (Different From the Others), a film that Hirschfeld had co-created with the Austrian director Richard Oswald (Weber and Burgmair, 1997: 13). Here, Wilhelm accused Kraepelin of making the very same mistake himself. What Kraepelin described as the ‘promotion of homosexuality’, Wilhelm, in his own words, described as something else entirely: ‘Kraepelin’s writing suggests that he understands such a “promotion of homosexuality” to encompass every gathering of homosexuals amongst themselves, be it for social reasons or to defend themselves against defamation’ (Praetorius, 1922: 43). For Wilhelm, the claim that homosexuality could be promoted was illogical, because homosexuality was inborn. By collapsing human and butterfly ‘nature’ and fixing the origin of homosexuality in the body through reference to Goldschmidt’s butterfly experiments, he reclaimed homosexual culture in the form of homosociality and mutual support against discrimination.

This move to depoliticise nature and instead cast a light on the retrograde politics of those who considered homosexuality to be pathological or acquired appeared in another review, in which Wilhelm criticised an article published by Gustav Fritsch, an anatomist, anthropologist, and neuropsychiatrist based in Berlin, on the ‘so-called third sex’ of humans. Fritsch, referring to both human and non-human animal organisms, argued that two sexes always appeared fully differentiated, and that even in cases where sex organs appeared hermaphroditic, this did not prove a bisexual disposition. Rather than assume an originary bisexuality, Fritsch suggested we should simply speak of ‘monstrosities’, the term denoting an unnatural abnormality of growth, a deviation from the norm of male and female sex. Wilhelm’s review of Fritsch’s article followed the typical pattern of his Bibliography, as outlined by Dubout: He heavily criticised Fritsch’s article, which argued against the bisexual predisposition of humans, and instead proposed the usual argument that homosexuality and other sexually intermediate conditions were inborn (Dubout, 2016). In order to prove Fritsch wrong, Wilhelm enlisted Ernst Haeckel’s work on gonochorism (sexual separation into two distinct sexes) versus hermaphroditism, which showed the prevalence of hermaphroditism in the animal kingdom and in humans, to argue that the primary disposition was clearly hermaphroditic; Eugen Steinach’s work on gonadal transplantation experiments to argue that transplanted sex glands could bring out a change in sexual behaviour only if the predisposition of that sex was already present; and finally, Goldschmidt’s butterfly experiments, arguing that these confirmed once and for all the hermaphroditic early stages of both butterflies and humans. Here, he made particular reference to Goldschmidt’s article on human intersex, arguing that Goldschmidt’s research showed that a quantitative system of potency decided which characteristics were latent and which manifest. For Wilhelm, this had particularly clear consequences for understanding nature’s ‘intent’:

The apparent proof, which Fritsch deduces from the supposed tendency of nature towards sexual differentiation, is fairly weak, because we do not know which tendency nature pursues and, vice versa, we can deduce from the hermaphroditic living things which, without a doubt, exist, that nature has a tendency to bring forth a so-called Third Sex for some metaphysical purpose. (Praetorius, 1919: 177)

What he was questioning here was the ‘nature’ of nature. If nature brought forth intermediate sexual stages, it was nonsensical to call these individuals monstrous, that is to say unnatural. As in his review of Kraepelin’s work, this then led to a criticism of Fritsch’s misguided *politics*: Wilhelm voiced his suspicion that Fritsch’s biological theories were really guided by Fritsch’s conviction that paragraph 175 of the German Criminal Code should be maintained. His scientific argument that sexual intermediacy could not be linked to physical traits, Wilhelm argued, served this political purpose. It was scientists of Fritsch’s ilk, not the gay rights activism of Wilhelm and his colleagues, that were driven by a political agenda.

In Goldschmidt’s butterfly experiments Wilhelm recognised a supporting argument for his ongoing research into the innateness and naturalness of homosexuality. In finding Goldschmidt’s experimental proof and listing it next to the work of Haeckel and Steinach, Wilhelm built a case for the naturalness of homosexuality. In doing so, Wilhelm depoliticised nature and instead revealed its interpreters, such as Kraepelin and Fritsch, as driven by political goals. Goldschmidt, who was not associated with the group of sexologists centred on the SHC or the Institute of Sexology, and who nonetheless argued that intersexuality occurred in nature, also served as a suitable politically neutral person to underline the point that Hirschfeld and Wilhelm, who argued against the decriminalisation of homosexuality, were not politically driven, but that Kraepelin and Fritsch were. As a result, for Wilhelm’s – and, by extension, Hirschfeld’s – sexological project, Goldschmidt’s butterflies were politically potent.

## Heredity and psychology: Arthur Kronfeld

Arthur Kronfeld (1886–1941) was a psychiatrist and founding member of the Institute of Sexology in Berlin, where he worked from 1919 to 1926 (Sigusch, 2008: 359). In 1922, the *Yearbook* published Kronfeld’s article ‘Zu den Problemen der Konstitution bei der Homosexualität’ (Regarding the Problem of the Constitution of Homosexuality), which evaluated the contributions of *Konstitutionsforschung* to the study of homosexuality (Kronfeld preferred the term *Gleichgeschlechtlichkeit*, meaning *same-sex-ness*). The term *Konstitution* was taken from the context of the biological sciences. In the context of the study of sexual constitution in sexology, it was used synonymously with the term *Anlage*, or *(pre)disposition*. In his dictionary of sexology, Max Marcuse’s entry on *Sexualkonstitution* acknowledged that, although research in this area was unified in the sense that it agreed that sex was founded in certain predispositions of the organism, there was no agreement on the scale or depth of these predispositions, their variations, or their interdependence (Marcuse, 2001[1926]: 720). Kronfeld’s use of Goldschmidt’s evidence posited biological explanations for sexual diversity at the root of individual development and ‘constitution’, but his article also acknowledged other influences, such as drives, desires, and psychological and social factors, on the development of sexuality. This attempt to reconcile a biologistic model of sexuality with a psychological model posed particular political difficulties.

In his article, Kronfeld listed what he considered to be two recent scientific developments that contributed to the understanding of the constitution of homosexuality: the discovery of hormones and the study of heredity. As Nelly Oudshoorn shows, once the concept of sex hormones had been developed after 1900, sex researchers quickly came forward to show the shared responsibility of hormones and genes for sexual development (Oudshoorn, 1994: 20–1). Sexual development itself was broken down into two stages: that of sex determination, demarcated as the realm of genetics, and that of sexual differentiation, the realm of endocrinology (ibid.: 21). Kronfeld’s interest in both hormones and the study of heredity therefore indicated a holistic interest in sexual development more broadly, rather than sex determination or differentiation specifically.

Kronfeld went on to argue that *Konstitutionsforschung* consequently put forward three hypotheses: that the development of sex depended on substances that affected a physiological result (namely hormones); that the production of these substances could be traced back to the constitutional foundations of the organism; and that this constitutional foundation could be traced back to the hereditary material of both parents. However – and crucially – Kronfeld did not argue that these findings and the addition of endocrinological and genetic research would be able to explain the homosexual constitution in its entirety. The interplay between hormones and hereditary material, Kronfeld argued, meant that sexuality was variable, and that fluid transitions existed between fully male and female types. In this, he acknowledged Hirschfeld’s theory of sexual intermediacy, which he accepted as a valid theory, but not one that had been proven empirically or experimentally. In this instance, Goldschmidt’s butterfly experiments were not listed as evidence, because they did not seem to deliver the appropriate proof (as Wilhelm and Hodann believed) that sexual intermediacy existed. However, they were mentioned separately to offer one piece of evidence in a much larger puzzle that *could* explain homosexuality, an explanation that Kronfeld did not believe had been fully developed. Ulrike Klöppel notes that Kronfeld understood Goldschmidt’s term *intersex* as a simply descriptive term in this context, and did not acknowledge Goldschmidt’s genetic explanation of intersexuality with regard to humans. Kronfeld’s complaint that Goldschmidt’s theory of intersex was too one-sided, and focused on genetic traits whilst neglecting epigenetic and endocrine factors of sex differentiation, was a simplification of Goldschmidt’s theory, which did indeed acknowledge the importance of the inner secretions (hormones) on sex development. As Klöppel notes, in various publications Goldschmidt was presented as a fierce proponent of a theory of chromosomes and their importance, alongside Morgan, despite huge differences between the two (Klöppel, 2010: 415). In actual fact, however, Goldschmidt’s theory of sex determination indeed acknowledged the importance of hormones in ‘higher animals’ (Goldschmidt, 1917), and thereby resisted the reductionism prevalent in US genetics, which reduced phenotypic traits to genetic causes only (Klöppel, 2010: 409).

Unlike Hodann and Wilhelm, Kronfeld displayed a much more critical stance on Hirschfeld’s theory of sexual intermediacy. Kronfeld added that most cases of homosexuality could not be matched exactly to laws of nature discovered by constitutional biology, because homosexuality stood in intimate relation to an individual’s psychological and social personality. Kronfeld was not entirely satisfied with Hirschfeld’s theory, because he considered it to be too biologistic (Herzer, 2017: 301). In his *Sexualpsychopathologie*, published only a year after the article in the *Yearbook* discussed here, Kronfeld proposed an approach to the study of sexual variation through the lens of sexual psychopathology, which would bring together biological and psychological explanations for homosexuality (same-sex-ness).

In the conclusion to his article, Kronfeld argued that ‘same-sex-ness is connected with the very being of its carriers, it is fatefully connected with its constitution; it is not a coincidental perversion of the soul and the drives…but is a necessary and deep-seated desire of being [*Wesensbedürfnis*] in the foundation of the whole individual’ (Kronfeld, 1922: 30). Here, Kronfeld was clearly outlining an understanding of homosexuality that required knowledge of an individual’s constitution (hormones and hereditary material), and rejecting approaches to the study of homosexuality as acquired perversion. An individual’s constitution, he argued, drove that individual’s desires in a manner resembling fate, but he also asserted that drives, desires, and the psychological and social personality of an individual were factors that indicated an individual’s sexuality, and should therefore be examined more closely. For Kronfeld, Goldschmidt’s butterfly experiments showed that some part of homosexuality was linked to an individual’s disposition, potency, and potential, but that sexuality was essentially dynamic. Consequently, butterfly intersex could not provide a model, merely a basis. Goldschmidt’s butterfly experiments were able to contribute knowledge about hereditary factors of sexuality, because the rules of heredity applied to butterflies and humans alike, but they did not contribute to our knowledge of psychological and social factors, and it was these that Kronfeld determined to be an important and dynamic part of an individual’s sexuality.

As in the work of Hodann and Wilhelm, sexual intermediacy here was linked to the constitutional core of one’s being, but unlike Hodann and Wilhelm, Kronfeld proposed other equally important factors that influenced sexual development. This approach was only partially reconcilable with a sexual rights programme that had been established on the very basis of biological explanations for homosexuality. As a consequence, Kronfeld’s understanding of sexuality, which clearly acknowledged the constitutional force of an individual, but also sought to explore psychological factors, and did not give the same amount of credit to Goldschmidt’s butterflies as did Wilhelm and Hodann, would eventually lead to a break from Hirschfeld. In 1926, Kronfeld left the Institute of Sexology and opened his own psychiatric practice in Berlin.

## The laws of nature and their limits: Arthur Weil

In 1923, Arthur Weil (1887–1969) published an article in the *Yearbook* entitled ‘Geschlechtsbestimmung und Intersexualität’, which made renewed use of Goldschmidt’s butterflies. Weil was a veterinarian, physician, and endocrinologist and a colleague of Hirschfeld and Kronfeld, who worked at the Institute of Sexology from 1921 to 1923. Weil had worked with Emil Abderhalden, who, in 1912, developed a blood dialysis method that would later be developed to serve as a pregnancy test (Sigusch and Grau, 2009: 736). Sexologists interested in endocrinology, including Hirschfeld and Bloch, thought that this test could be used to detect homosexuality; Hirschfeld asked members of the SHC to donate blood samples to test this procedure (ibid.). After Hirschfeld failed to attract the famous Viennese endocrinologist Eugen Steinach to join his Institute, he hired Weil in 1921 as director of the Abteilung für innere Sekretion (Department for the Study of Inner Secretions; Herrn, 2004). Weil stayed in this role for a brief period only, travelled to the United States two years later, and did not return. As I argue in this section, Weil used Goldschmidt’s butterflies as reliable evidence to prove certain laws of nature that also apply – albeit in different ways – to humans, and therefore offered a measurable, empirical approach to the study of human sexuality that was more easily incorporated into the sexual rights agenda represented by the Institute of Sexology.

Weil began his article by outlining the recent contributions of research in genetics to determine the inheritance of sex. Whilst experiments were often based on animal model organisms, Weil argued that the results of these experiments were relevant to the study of the human *Geschlecht*, too. What Weil was interested in discovering was a ‘natürliche Gesetzmäßigkeit’, the ‘laws of nature’ ruling the prevalence of sexual intermediacy (‘Komitee-Mitteilungen’, 1922: 108). Following Goldschmidt, Weil argued that it was possible to use research on butterflies to make statements about humans once the different rules that applied to each class of animals had be determined. In this respect, Goldschmidt’s butterflies simultaneously revealed continuity and discontinuity between humans and butterflies: The same rules of heredity applied, but insects and humans also followed different laws of nature. Whereas in insects, sex was fixed once egg and sperm had met to form a fertilised egg, and the development of sex happened independently from hormones, in humans hormones had a significant influence on the sexual development of the organism. Weil wanted to know which forms of sexual intermediacy were caused by the parental predisposition, and which by hormonal levels. And here he argued that Goldschmidt’s work on butterflies and his statement on potency and valence could be used to draw conclusions for humans, but that humans had the additional influence of hormones. As one example, he mentioned the development of breasts: Some men with otherwise typical physical development had what appeared to be female breasts. These individuals, Weil argued, were results of the cross-breeding of two parents with different sex valence figures, just like Goldschmidt’s moths. As such, breasts developed in a female way until male hormones influenced the organism in a male direction (Weil, 1923: 34f).

Goldschmidt’s butterflies, Weil asserted, served as partial, but foundational evidence to uncover the laws of nature that determined the development of sex/sexuality in humans. Yet this reference to the natural laws, or laws of nature, is somewhat opaque, because it is not entirely clear whether Weil was using ‘nature’ to mean non-human animals, or individuals (whether human or non-human) ‘in the wild’ (as opposed to the laboratory). ‘In nature’, Weil argued, ‘one can often find, for example in butterflies such intersex forms’ (Weil, 1923: 29). Yet these intersex butterflies, which Weil claimed were to be found in nature, were actually found in Goldschmidt’s controlled laboratory environment. Weil referred to the ‘nature experiment’ – Goldschmidt’s butterflies – to argue that in the case of the butterfly, hormones played no important role. ‘Nature’ here referred to intersex butterflies bred in the carefully controlled space of the laboratory; what was natural about them was their animal biology. The laws of nature Weil sought to uncover, then, applied to non-human animals fully, but humans only partially, because of the ‘Höherentwicklung’ (‘higher development’ in evolutionary terms) of the latter (ibid.).

In a way similar to Kronfeld, Weil argued that butterfly experiments could illuminate the very core and origin of the human *Geschlecht*, the foundational unit of the sexed organism, but they were also unable to explain the human *Geschlecht* fully. The reasons for this, however, were very different for Kronfeld and for Weil. Whereas Kronfeld found a fuller explanation of the human *Geschlecht* in the psyche, Weil found it in another aspect of the human body: the hormones secreted from the sex glands. In both cases, butterfly experiments became a central and pivotal piece of the sexological puzzle that was made to fit neatly into a pre-existing sexological theory. In the case of Weil, butterfly experiments served to illuminate the innermost aspects of the human *Geschlecht*, the genes, and the union of the miniscule sperm and egg, in order to explain physical differences that could be read from the body’s surface, for example the anomalous development of breast tissue in human males. Most importantly for a discussion of the sexual politics of animal evidence, however, is that Weil’s composite hereditary-hormonal approach to the study of sex/sexuality was more easily incorporated into the sexual politics of sexology, because it offered more measurable results and a clear ‘law of nature’.

## Nature and artificiality: Magnus Hirschfeld

In 1923, Magnus Hirschfeld (1868–1935) published an article in the *Yearbook* that commented on how Goldschmidt’s butterfly experiments fitted into his own sexological project. The article was an extended version of a talk on intersexual constitution that he had originally presented at a conference with specific focus on the relationship between constitution and sexuality at the Hygiene Institute of the University of Berlin on 16 March 1923 (Hirschfeld, 1923: 3). In this article, Hirschfeld offered a summary of the history of the study of human intersexuality:

When I published the first volume of the *Yearbook of Sexual Intermediacy* 24 years ago, the study of human intersexuality was still relatively unknown and did not strike a responsive chord. What today appears as obvious, almost banal, appeared as grotesque and stubborn unilateralism. Back then, neither the biological works of Neugebauer, Tandler and Steinach, nor the sensational works of Fließ, Freud and Weininger had been published, and the experiments of cross-breedings, conducted by Morgan, Goldschmidt and others had not begun. Gregor Mendel’s rules of heredity were still ignored and Brown-Sequard’s thoughts on inner secretion were not taken seriously; the concept of sexology itself was almost as unknown as its name. (ibid.: 8)

In this, Hirschfeld acknowledged the huge shift in thinking about human intersex (which he here used synonymously with *sexual intermediacy*) that had happened between 1899 and 1923. Hirschfeld recognised that the work of Franz von Neugebauer (on hermaphroditism), Julius Tandler (on public health and eugenics), Eugen Steinach (on the endocrine system), Wilhelm Fließ (on innate bisexuality), Sigmund Freud (whose psychoanalytic theory had shown that homosexuality was not a random quirk, but followed specific psychodynamic rules), Otto Weininger (on innate bisexuality), Thomas Morgan (on the heredity of sex), Richard Goldschmidt, Gregor Mendel (credited with discovering the rules of heredity), and Brown-Sequard (on rejuvenation through monkey gland experiments) together formed the definitive corpus of thinking about sex and sexuality in the context of constitution. This corpus included a wide variety of methodologies, from experiments (Goldschmidt, Morgan) to philosophical treatises (Weininger); and foci, from social systems (Tandler) to anatomical systems (Steinach), with a focus on a variety of organisms, from peas (Mendel), to butterflies (Goldschmidt), fruit flies (Morgan), and humans (Neugebauer).

By pointing out that his own research had taken place before the major scientific works on the study of sex had been produced, Hirschfeld was situating his own sexological work at the avant-garde of sex research, and claiming that the very idea that sex might be worth studying was only just coming into being. Hirschfeld claimed that his own work recognised intersex before the topic was discussed, before research in biology, psychoanalysis, genetics, zoology, and endocrinology had come to the same conclusions, and before sexology had started to be taken seriously as a discipline. Hirschfeld thus subsumed the studies of a whole generation of scientists from diverse disciplines and their scientific evidence to the sexological project.

Hirschfeld’s speech-cum-article continued:

When I drew up the study of sexual intermediate stages, I based this on purely clinical observations, but drawing on the broadest possible mass material, which did not come to me, but which I sought out. Each patient file collected in a consultation, even that of the most prolific psychiatrist, only shows a one-sided view. And as important as the findings of artificial breeding of bugs and butterflies may be, which have caused a deserved stir amongst geneticists, by far more value comes from studying the results of breeding which nature herself has done. (Hirschfeld, 1923: 9)

For Hirschfeld, the problem with animal experiments was not the difference in species, but the difference in scientific method. Hirschfeld’s criticism of Goldschmidt’s work on butterflies was that the conditions of his experiments were artificial. This can be seen from the fact that, in his list of contributions, Hirschfeld classified Goldschmidt as an experimenter rather than listing him as a biologist. In contrast, Hirschfeld considered his own ‘purely clinical observations’ of people in real-life situations less artificial. For Hirschfeld, observing the homosexual at a club or bar, or the ‘cross-dresser’ navigating metropolitan Berlin (he wrote a book about this in 1904: *Berlins Drittes Geschlecht*; Berlin’s Third Sex), was to observe nature in its natural habitat. Here, Hirschfeld revealed his fascination with a sociological approach to the study of sexual intermediacy. *Berlin’s Third Sex* was published in a series called *Großstadt-Dokumente* (Metropolis Documents), which presented a series of sociological studies of fringe and marginalised groups, including anarchists, homosexuals, sex workers, and professional musicians, and which covered such topics as religious sects, gambling, and people smuggling (Jazbinsek and Thies, 1997: 45). For Hirschfeld, publishing a book about homosexual subculture in Berlin was certainly a *scientific* endeavour. In *Berlin’s Third Sex*, he wrote, ‘It is certainly prudent that the presentation of such a difficult issue in a popular science setting be left to those who have attained the necessary qualifications and skills by virtue of extensive scientific research and experience’ (Hirschfeld, 2017[1904]: 9). He was making the claim here that sociological study was certainly commensurable with the scientific study of sexual intermediacy, more so than experimental work in an artificial setting.^12^

In contrast to the sociological study of sexual intermediate life, Hirschfeld implied, Goldschmidt’s butterflies lived, bred, and died as experimental objects in the lab, and the conditions of their life should therefore be considered less natural and of lower scientific value. Goldschmidt’s experimental studies, which were ‘artificial’, provided useful scholarship to draw upon, but these artificial experiments needed to be advanced through sexological observation ‘in nature’. Here, Hirschfeld’s approach showed a significant difference from Weil’s understanding of nature as animal biology. Hirschfeld's argument also exhibited an interesting inversion of the natural and the artificial. Hirschfeld saw a hierarchy between the study of heredity by way of experiments and that by way of clinical observation, a hierarchy or rivalry that, for example, Hodann did not recognise in his article from 1917.

Yet Hirschfeld clearly did find experiments on butterflies useful. In a report on the activities at the Institute during its first year in 1919, the following statement appears:

During its first year, the actual experimental work did not advance, due to the difficult external conditions.…We also began the breeding of exotic silk-moths (*Attaccus orizaba* and *Actias luna*) in order to conduct bastardisation experiments and achieve intersex variations through cross-breeding. However, none of this experimental work should be considered as finished at this point. (Hirschfeld, 1920: 65–6)

**Figure 3.**
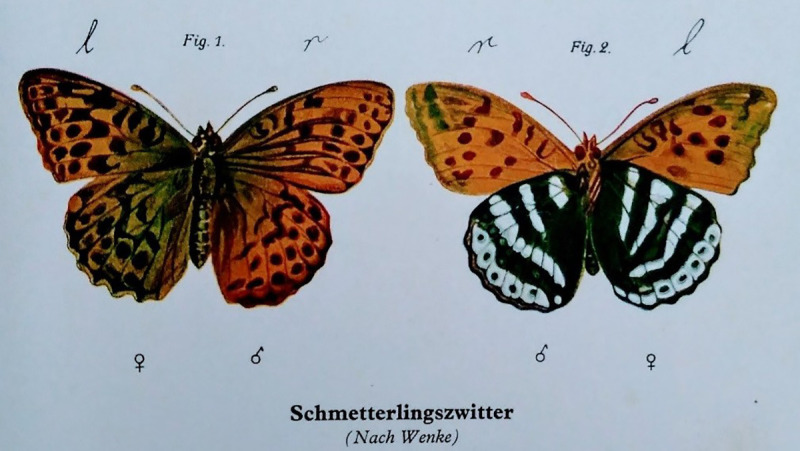
‘Schmetterlingszwitter’ (hermaphroditic butterflies; Hirschfeld, 1930: plate 33).

In its first year of activity, and despite the political upheaval in Germany following the recent war, Hirschfeld and his colleagues were investing time into breeding moths and butterflies. They did so because they thought that it added to their knowledge about sexual intermediacy. Much like a lepidopterist who collects butterflies, Hirschfeld collected methods from a large variety of disciplines. Figure 3 shows an image taken from *Geschlechtskunde* (the same volume where the image of *Perrhybris lypera Magni Hirschfeldi* also appeared) of hermaphroditic butterflies found on Hasselwerder Island near Lake Tegel in Berlin, only six miles from where Hirschfeld lived. This is not a coincidence: Hirschfeld was deeply interested in exploring homosexuality in its natural habitat, and he may have had particular interest in local sexually-intermediate butterflies caught ‘in nature’. Comparing lab-bred butterflies to the homosexual population of Berlin could also have been interpreted to imply a sense of breeding and purpose in homosexuality; strategically, Hirschfeld might have found it more suitable to find butterflies in the wild to support his view that homosexuality was naturally occurring, rather than a cultural phenomenon. The influence of intersex butterflies on Hirschfeld’s sexological work and the non-human animals he became interested in was so pronounced that butterflies not only took up residence in his Institute and featured in his major publications, but also became a symbolic representation of his sexological achievements when, in 1929, the *Perrhybris lypera Magni Hirschfeldi* was named in his honour.

## Conclusion: ‘The image of a butterfly coming to life’

In this article, I have explored how Goldschmidt’s experimental work on intersex butterflies was marshalled as evidence to illuminate the nature and naturalness of ‘sexual intermediacy’, in particular homosexuality, by sexological writers between 1917 and 1923, and how butterfly evidence informed the sexual politics of the group of sexologists affiliated with the Institute of Sexology. Hirschfeld’s sexology exchanged concepts with cutting-edge life sciences. In considering the history of the specific significance of intersex butterfly experiments in the sexological context, this article was not intended to judge retrospectively the successes or failures of certain scientific claims, but to show that this interdisciplinary interest that extended to neighbouring disciplines and their research on non-human animals was an integral part of sexology’s public profile during the interwar era. This history speaks to sexology’s dual life as a set of authoritative pronouncements (backed by biological expertise) and a countercultural endeavour to undercut existing norms.

Intersex butterflies functioned as important and politically charged evidence for a discussion at the heart of the sexological project of those involved in the founding of the Institute of Sexology: the question of the nature and naturalness of homosexuality (and sexual intermediacy more broadly) and its political consequences. Goldschmidt’s butterflies fluttered through the work of each of the sexologists discussed here, and provided evidence for their various theories. Their significance is far from obvious. Butterfly evidence showed a level of plasticity, as each sexologist incorporated Goldschmidt’s butterflies in different ways and in different stages of human sexual development: Hodann used the results of butterfly experiments to provide clinical applications; Wilhelm depoliticised nature and firmly ascribed bad politics to Hirschfeld’s opponents; Kronfeld only partially accepted their authority, but pushed beyond constitution to psychology; Weil looked to butterflies to understand universal laws of nature; and Hirschfeld criticised the artificiality of the butterfly experiment. At the same time, the use of butterfly evidence in the work of the sexologists discussed above shared a common reference point: the idea that non-human animals and humans were connected through a shared investment in a concept of sexual nature. Each of the intellectual intermediaries discussed in this article accepted that intersex butterflies could tell them something about human sexual variation. Goldschmidt’s intersex butterflies provided a potent tool for understanding sexuality in the human realm and for drawing sexual-political conclusions. These butterflies were also political actors in the history of sexology, offering a way of figuring out where people’s allegiances lay and how they fitted into a specific sexological vision that argued for a biologistic model of sexual intermediacy.

**Figure 4.**
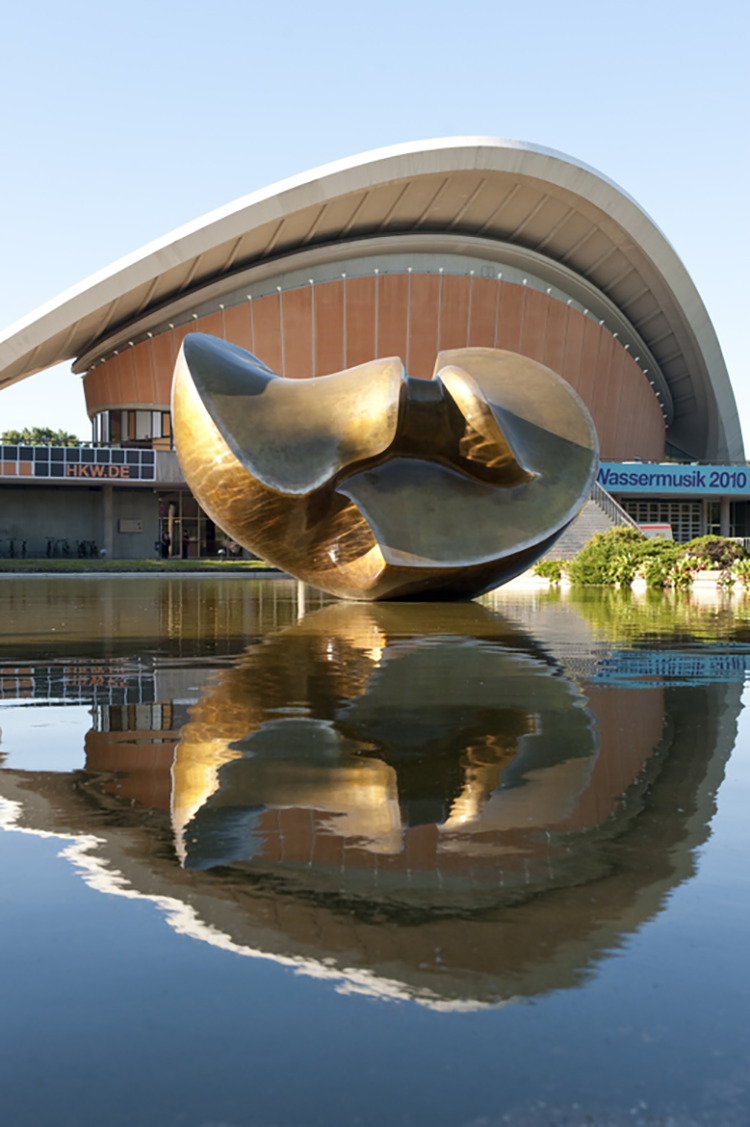
‘Large Divided Oval: Butterfly’, Henry Moore, 1987, Photo: Sebastian Bolesch/HKW, 2010 (Haus der Kulturen der Welt, n. d.).

Although the importance of these butterflies for sexology has so far been neglected in scholarship on the history of sexology, traces of these butterflies remain. From 1919 to 1933, the Institute of Sexology stood at the corner of Beethovenstraße 3 and In den Zelten 10, close to the river Spree and in the heart of Berlin. After the Institute was ransacked in 1933 and destroyed in World War II, a conference centre was built on the site. Today, this is the Haus der Kulturen der Welt (HKW), the House of World Cultures. Here, in the vicinity of Hirschfeld’s former Institute, a sculpture by Henry Moore was erected in 1987 (Figure 4). The sculpture, a large bronze-coloured object positioned in the middle of a shallow pond, is called ‘Large Divided Oval: Butterfly’. The website of the HKW describes it thus:

The play of light and reflective water makes the sculpture appear as if it is opening upwards and expanding beyond the borders of its form. It plays with the impression of being a pair of wings, thus awakening most intriguingly the image of a butterfly coming to life.

The presence of Moore’s butterfly sculpture where the Institute of Sexology once stood is a coincidence, but a fateful one. After the rise of National Socialism in Germany and the destruction of the Institute, German sexology ceased to exist in the way it had at the height of its success, and sexological research shifted to different geographic regions (as Katie Sutton’s article in this special issue shows). It was not until the 1980s that the history of sexology in Germany was taken up as a subject of study, most notably through the Magnus Hirschfeld Gesellschaft in West Berlin. Since then, the history of sexology has become a topic of increased interest and has, as this special issue shows, diversified into multiple histories of sexology. It is hoped that this article has contributed another history of sexology, one that ‘awaken[s] most intriguingly the image of a butterfly coming to life’ and makes a case for paying attention to non-human actors in the interdisciplinary history of sexology.

## Notes

I presented the early stages of this work at the British Animal Studies Network meeting on the topic ‘sex’ at the University of Strathclyde and the Animals and Society Institute at the University of Illinois at Urbana-Champaign, and am grateful for the comments and feedback that I received there. I am extremely grateful to my anonymous reviewers and to the special issue editors, Katie Sutton and Kirsten Leng, for their careful reviews of this article. Their comments and feedback greatly improved my argument. I would like to thank the Rethinking Sexology team (Kate Fisher, Jana Funke, Jen Grove, Sarah Jones) and Kazuki Yamada for their comments on this article and support throughout. I also thank the Haus der Kulturen der Welt for permission to reproduce Figure 4.

1. The title *Geschlechtskunde* is often translated as *Sexual Science*. This might cause confusion, as it translates back into German as *Sexualwissenschaft*. A more appropriate translation of *Geschlechtskunde* might be *Sexual Studies*.

2. Whilst this article focuses on the reception of Goldschmidt’s intersex butterflies in the *Yearbook*, these butterflies were discussed more widely, for example by Agnes Bluhm in her article on sex determination in Marcuse’s (2001[1926]) *Handwörterbuch der Sexualwissenschaft* (Concise Dictionary of Sexology). In 1922, Kurt Wolff submitted a dissertation to the Friedrich Wilhelm Universität zu Berlin in which he tested Goldschmidt’s hypothesis on intersexuality using material from the Institute of Sexology, where he worked sometime between 1921 and 1922 (Wolff, 1922). Outside of Germany, the Spanish sexologist Gregorio Marañón referred to Goldschmidt’s work on intersex butterflies (Cleminson and Vázquez García, 2009: 156, note 143).

3. This article refers to butterflies, rather than moths, throughout. The taxonomical order of lepidoptera, belonging to the class of insects, contains both butterflies and moths. In German, lepidoptera are confusingly referred to as *Schmetterlinge*, meaning *butterflies*, even though as a taxonomical order it refers to both butterflies and moths. Throughout this article, I will be referring to butterflies, rather than moths, to represent the terminological ambiguity of the German sources. This is important because in this article I not only want to trace Goldschmidt’s experimental work on *Lymantria dispar* (order: lepidoptera, sub-order: moths), but am predominantly interested in the sexual-political meaning of his work for sexologists around Magnus Hirschfeld. I want to leave space for the possibility that sexologists were interested not only in the scientific findings of Goldschmidt, but also in the affective pull and the representational power they exerted.

4. For the history of the fruit fly as an experimental organism, see Koehler (1994).

5. For the connection between Goldschmidt’s work and the larger social and political climate, see Dietrich (2000); Ha (2011); Koehler (1998); Satzinger (2009a, 2009b).

6. In several cases, Goldschmidt tried to respond to these uses of his work. In 1927, he wrote about the ‘mixed-race’ inhabitants of the Japanese Bonin Islands (today Ogasawara Guntō), arguing definitively that ‘cross-breeding’ on the islands had not had a degenerative effect (Satzinger, 2009b: 167–9). Satzinger also argues that Goldschmidt’s evolutionary theory did not rely on a concept of race as an essential characteristic, and that his later work avoided using the term (ibid.: 166). Despite this, Satzinger also notes that ‘Goldschmidt certainly saw breeding and eugenic applications as possible. In the 1920s, he was involved in the drafting of a law on voluntary eugenic sterilization’ (ibid.: 155).

7. Homosexuality was frequently understood on the basis of a biologistic model, which sought to find the origin and presentation of homosexuality in the body and its expression (see Steinach’s endocrinological model discussed above). It was therefore possible for Goldschmidt and others to argue that homosexuality was a form of intersex. In 1931, Goldschmidt withdrew the claim that homosexuality was a form of intersex (Goldschmidt, 1931). The reasons for this are complex and cannot be discussed within the scope of this article.

8. Ellis also compared sexual inversion to ‘coloured-hearing’, or synaesthesia (Ellis and Addington Symonds, 2008[1896]: 204).

9. Moll was an influential sexologist who advocated for apolitical and impartial science. For more on Moll, see Sigusch (2012).

10. All translations are my own, unless otherwise noted. The German term *Wissenschaft* is not identical to the English-language term *science*, but includes the arts, humanities, and social sciences as well as the natural and medical sciences. Goldschmidt's comments should therefore not be read as a comment on the natural sciences alone, but as a broader comment on interdisciplinarity across the social sciences, humanities, and natural sciences.

11. Although extensive inquiries were undertaken, it was not possible to determine the copyright holder for this image. Goldschmidt’s works are now available in the public domain via www.archive.org.

12. Josch Hoenes similarly notes that Hirschfeld’s use of butterfly images in *Geschlechtskunde* shows that his sexological method relied on visual observation of – rather than experimentation with – nature and life. Hoenes puts this down to Hirschfeld’s interest in monism, which rejects dualisms of body and soul, as well as nature and culture, and promotes holistic thinking (Hoenes, 2019: 79f).
